# Supplementary material for: An evaluation of the early impact of the COVID-19 pandemic on Zambia’s routine immunization program
Source: PLOS Glob Public Health. 2023 May 2;3(5):e0000554. doi: 10.1371/journal.pgph.0000554 (PMC10153718; doi:10.1371/journal.pgph.0000554)
Supplement: S1 Text — (PDF) [file pgph.0000554.s001.pdf]

An evaluation of the early impact of the COVID-19 pandemic on Zambia's vaccination program

S1 Text. Estimating pre-pandemic routine vaccination coverage for MR1, Penta1, and Penta 3

We relied on data on routine vaccination coverage from Zambia's 2018 cross-sectional Demographic and Health Survey (DHS) made publicly available by ICF International to estimate the lifetime probability and monthly rate of receiving routine vaccination for each district in Zambia. The national DHS survey has one record for each interviewed woman's child aged 0 to 36 months at the time of the survey (N=5,670), and is linked to a database of GPS coordinates (latitude and longitude) of respondents' home locations. The coordinates are aggregated on the scale of clusters containing approximately twenty households, and randomly jittered up to 2 kilometers in urban areas and up to 5 kilometers in rural areas to protect respondent confidentiality (however, the jittered coordinates remain within their respective administrative districts). Children included in the 2018 DHS survey were too young to have been eligible for vaccination in the most recent measles supplementary immunization activity (SIA) campaign conducted in 2016, so we focus here on routine vaccination only.

For each child, we extracted from the DHS: age at the time of survey, whether the child had ever received a MR1, Penta1, and Penta3 vaccine (each based on either vaccination card or report of parent/guardian), and age at the time of the vaccine (if a vaccination card was available). Data is available from 112 of the 116 designated districts in 2018. For MR1, Penta1, and Penta3, children under 8, 0.5, and 2.5 months of age at the time of DHS survey, respectively, were excluded from the analysis; they were considered not to be "at risk" for vaccination since recommended MR1 administration is at 9 months, 6 weeks, and 14 weeks age, respectively, in Zambia. We modified a survival analysis approach developed in [1,2] to estimate parameters associated with vaccination coverage. A child with a vaccination card was considered to be uncensored at the time of the vaccination (we assume that vaccinations recorded on cards only represent routine vaccination). When a parent reported that their child had received vaccination but a vaccination card was unavailable, the child was considered to be left censored at the time of DHS survey; a child reported as unvaccinated was considered to be right censored at the time of DHS survey.

For an individual j at district i, the probability that this individual has been vaccinated depends on three classifications of individuals: uncensored ($u_{ij} = 1$), left censored ($l_{ij} = 1$), or right censored ($r_{ij} = 1$):

$$f(v_{ij};\theta_i) = Pr(T = v_{ij}) = \lambda_i \cdot e^{-\lambda_i \cdot (v_{ij} - m)} \quad \text{(Eq. 1)}$$

$$F(t_{ij};\theta_i) = Pr(T \leq t_{ij}) = p_i\left(1 - e^{-\lambda_i \cdot (t_{ij} - m)}\right) \quad \text{(Eq. 2)}$$

$$S(t_{ij};\theta_i) = Pr(T > t_{ij}) = 1 - F(t_{ij};\theta_i) \quad \text{(Eq. 3)}$$

T is a continuous random variable representing the age in months at receipt of vaccine; v_{ij} is the age in months at the receipt of vaccine, only available for uncensored individuals ($u_{ij} = 1$); t_{ij} is the age in months of an individual at the time of the DHS survey; p_i is the location-specific lifetime probability of being vaccinated through routine vaccination (e.g., the saturation parameter); λ_i is the location-specific rate of receiving the vaccine through routine vaccination after *m* months of age; *m* is vaccine-specific and is 8 for MR1, 0.5 for Penta1, and 2.5 for Penta3. The probability of routine vaccination reaches p_i more slowly (e.g., at an older age) for lower values of λ_i than it does for higher values (S2 Fig).

The parameters of interest, transformed for stability, are: $\theta_{1,i} = logit(p_i)$, and $\theta_{2,i} = log\ (\lambda_i)$. The likelihood of the parameters given the observed age and vaccination status of an individual is:

$$L(\theta_i; t_{ij}, v_{ij}, u_{ij}, r_{ij}, l_{ij}) = f(v_{ij};\theta_i)^{u_{ij}} \cdot F(t_{ij};\theta_i)^{u_{ij} + l_{ij}} \cdot S(t_{ij};\theta_i)^{r_{ij}} \quad \text{(Eq. 4)}$$

To estimate $\theta_{x,i}$ we assumed that it is multivariate normally distributed with a location-specific mean of $\mu_{x.i}$ (Eq. 5) and a conditional autoregressive model (CAR) specification for the spatial random effects, parametrized by the precision matrix $1/\sigma_x^2$:

$$\theta_{x,i} \sim Normal(\mu_{x,i}, \sigma_x^2) \quad \text{(Eq. 5)}$$
$$1/\sigma_x^2 = \tau_x(D - \alpha_x W) \quad \text{(Eq. 6)}$$

Where $1/\sigma_x^2$ is the precision matrix, $\tau_x$is a precision parameter, $\alpha_x$controls the spatial dependence (α=0 implies spatial independence, and α=1 collapses to an intrinsic conditional autoregressive model), D is an *i* by *i* diagonal matrix with diagonal elements encoding the number of adjacent neighbors that each district has, and W is a binary adjacency matrix. Because the distribution of the conditional autoregressive model is multivariate normal, we only include a spatial random effect in these models.[3]

References

1. Lessler J, Metcalf CJE, Grais RF, Luquero FJ, Cummings DAT, Grenfell BT. Measuring the performance of vaccination programs using cross-sectional surveys: A likelihood framework and retrospective analysis. PLoS Med. 2011 Oct;8(10).

2. Takahashi S, Metcalf CJ, Ferrari MJ, Moss WJ, Truelove SA, Tatem AJ, et al. Reduced vaccination and the risk of measles and other childhood infections post-Ebola. Science. 2015 Mar 13;347(6227):1240–2.

3. Banerjee S, Carlin BP, Gelfand AE. Hierarchical Modeling and Analysis for Spatial Data. 2nd ed. New York: Chapman and Hall/CRC; 2014. 584 p.
