# Supplementary material for: An evaluation of the early impact of the COVID-19 pandemic on Zambia’s routine immunization program
Source: PLOS Glob Public Health. 2023 May 2;3(5):e0000554. doi: 10.1371/journal.pgph.0000554 (PMC10153718; doi:10.1371/journal.pgph.0000554)
Supplement: S2 Text — (PDF) [file pgph.0000554.s002.pdf]

An evaluation of the early impact of the COVID-19 pandemic on Zambia's vaccination program

S2 Text. Estimating measles susceptibility by age (4 to 49 years old) using a model fit to serological data

To estimate district-specific measles seroprevalence, we applied an approach developed in [1] where hierarchical spatial models were fit to data on individual measles seropositivity from an analysis of residual samples from a national HIV serosurvey (Zambia Population-Based HIV Impact Assessment) in Zambia in 2016 [1]. The nested serosurvey consisted of 9,852 blood samples collected from individuals one month to 49 years old. District-specific random effects were included in the hierarchical model based on a conditional autoregressive (CAR) specification, meaning that estimated seroprevalence at any given district was conditional on the estimated seroprevalence of neighboring districts. The models assumed a binomial probability distribution for seropositivity and a log odds link. Informative epidemiological and demographic model covariates included: individual level HIV status and age, and district and age specific measles vaccine dose 1 coverage and exposure to local measles outbreaks. There was no information about individuals' history of measles vaccination or measles infection from the nested serosurvey; this meant we had to rely on population level (i.e., district and age specific) covariates associated with history of measles vaccination and measles infection. Measles vaccine dose 1 coverage was estimated from Zambia DHS 2013 data. We defined exposure to an outbreak as any individual alive and living in a district with two or more measles-specific IgM positive cases reported within a year. Measles case data was collected by Zambia EPI program and was available since 2012. We also included interactions between HIV serostatus and age, as well as HIV serostatus and squared age. See [1] for additional model details. To project measles seroprevalence in year 2020 (for use in this analysis), data specific to modeled covariates from 2020 were combined with posterior estimates of model parameters.

We conducted leave-one-out cross validation analyses to evaluate the performance of the model to predict district and age-specific seroprevalence. In these analyses we left out each district or age in years, retrained the model with the smaller dataset, and then used the new posterior estimates of the parameter values to predict seroprevalence for the district or age originally left out (Fig. S7-S8). The model does well to predict seroprevalence for missing ages, but not as well to predict seroprevalence for missing districts. This finding is expected, given our reliance on the model district-specific intercept (gamma parameter) that captures variation not explained by our demographic and epidemiologic covariates. We do not extrapolate our model to estimate seroprevalence for any new districts. Our extrapolations for 2020 estimates of seroprevalence rely on the assumption that the underlying district-specific impact on seroprevalence (i.e., district-specific intercept) is constant from 2016 to 2020. As a result, we only evaluate the change in R effective due to routine vaccination disruptions in each district and do not evaluate the magnitude of R effective itself. We also present sensitivity analyses of the change in R effective to assumptions about population susceptibility in 2020 (Fig. S9).

We additionally compared estimated seroprevalence in 2018 for ages 9 to 36 months old to estimated immunity derived from vaccination (i.e., age and district-specific vaccination from the DHS estimates above taking into account vaccine effectiveness). We found generally good agreement between the two estimates; 95% credible intervals overlapped in at least half of the ages (>=14 of 28 age groups) in 96% of districts (108 of 112) and 95% credible interval of immunity (via DHS) overlapped with the median estimated seroprevalence in at least half of the ages (>=14 of 28 age groups) in 47% of districts (53 of 112) (Fig. S10). Given that estimates of immunity from coverage data only includes immunity due to vaccination, we would expect there to be good agreement in districts where there is little natural infection (i.e., Zimba district, Fig. S10B) and lower than seroprevalence in districts where natural infection may still contribute to immunity (i.e., Chitambo district, Fig. S10B). However, in the majority of districts (including Lusaka, Fig. S10B), estimates of immunity from DHS data are higher than seroprevalence. This may suggest that i) DHS data is inherently biased to capture children who access healthcare and vaccination, ii) immunity levels waned very quickly for vaccinated children or the EIA kit has a lower sensitivity than documented by the manufacturer, or iii) model fitting issues causes biases in one or both estimates. Although we cannot totally rule out that potential that vaccine effectiveness is much lower in Zambia

during this time for than other populations or that the sampling units for each survey differ across districts and represent drastically different populations within a district.

The final step to estimate district-specific seroprevalence is to weight the seroprevalence estimates by district-specific population characteristics. We created a new dataset with all possible covariate groupings. For example, one possible covariate grouping is district Chadiza, province Eastern, HIV negative, 7 years old, and with no exposure to a local outbreak. We estimated the probability of seropositivity for each covariate grouping across 2500 samples from parameter posterior distribution sets, taking into account both uncertainty of the mean parameter values and uncertainty in the sampling process. We then weighted the probability of seropositivity by each covariate grouping and sampled parameter set per age and district by the proportion of individuals in that covariate grouping to get 2500 estimates of seroprevalence for each age and district.

References

1. Carcelen AC, Winter AK, Moss WJ, Chilumba I, Mutale I, Chongwe G, et al. Leveraging a national biorepository in Zambia to assess measles and rubella immunity gaps across age and space. Sci Rep. 2022 Jun 17;12(1):10217.
