# Supplementary material for: An evaluation of the early impact of the COVID-19 pandemic on Zambia’s routine immunization program
Source: PLOS Glob Public Health. 2023 May 2;3(5):e0000554. doi: 10.1371/journal.pgph.0000554 (PMC10153718; doi:10.1371/journal.pgph.0000554)
Supplement: S3 Text — (PDF) [file pgph.0000554.s003.pdf]

An evaluation of the early impact of the COVID-19 pandemic on Zambia's vaccination program

S3 Text. Estimating R effective

To estimate outbreak risk we calculated the measles effective reproduction number (R_e) for each month and district using next generation methods taking into account age-specific susceptibility and mixing patterns [1]. The next generation matrix **W**, with elements w_{ij}, represents the expected number of individuals in the *i*th age class that are infected by an infectious individual in the *j*th age class upon introduction of measles virus into a totally susceptible population. The basic reproduction number, or the average number of individuals infected by a typical infected individual, is given by $R_0 = p(A)$ where $p(A)$ represents the dominant eigenvalue of matrix A. We initially calculate **W** by scaling age contacts extracted per [2] to a conservatively assumed basic reproduction number of measles of 12 (although estimates vary widely [3]), such that $p(\boldsymbol{W}) = 12$. To estimate R_e for each district *d* and month of disruption *m* we reevaluate the dominant eigenvalue of **W** after multiplied by the proportion of susceptible population,

$$R_{e_{dm}} = \rho \begin{bmatrix} w_{11}S_{1_{dm}} & \cdots & w_{1z}S_{1_{dm}} \\ \cdots & \cdots & \cdots \\ w_{z1}S_{z_{dm}} & \cdots & w_{zz}S_{z_{dm}} \end{bmatrix}$$

(Eq. 1)

where, $S_{k_{dm}} = S_k(dm) / N_k(d)$ is the proportion of susceptible population in age-group *k* specific to district *d* and month of disruption *m*. The size of the population in age-group *k* specific to district *d* ($N_k(d)$) was assumed constant throughout the year 2020 using Zambia Central Statistical Office estimates.

References

1. Diekmann O, Heesterbeek JA, Metz JA. On the definition and the computation of the basic reproduction ratio R0 in models for infectious diseases in heterogeneous populations. J Math Biol. 1990;28(4):365–82.

2. Prem K, Cook AR, Jit M. Projecting social contact matrices in 152 countries using contact surveys and demographic data. PLoS Comput Biol. 2017 Sep;13(9):e1005697.

3. Guerra FM, Bolotin S, Lim G, Heffernan J, Deeks SL, Li Y, et al. The basic reproduction number (R0) of measles: a systematic review. Lancet Infect Dis. 2017 Dec;17(12):e420–8.
