# Supplementary material for: An evaluation of the early impact of the COVID-19 pandemic on Zambia’s routine immunization program
Source: PLOS Glob Public Health. 2023 May 2;3(5):e0000554. doi: 10.1371/journal.pgph.0000554 (PMC10153718; doi:10.1371/journal.pgph.0000554)
Supplement: S1 Fig — (PDF) [file pgph.0000554.s004.pdf]

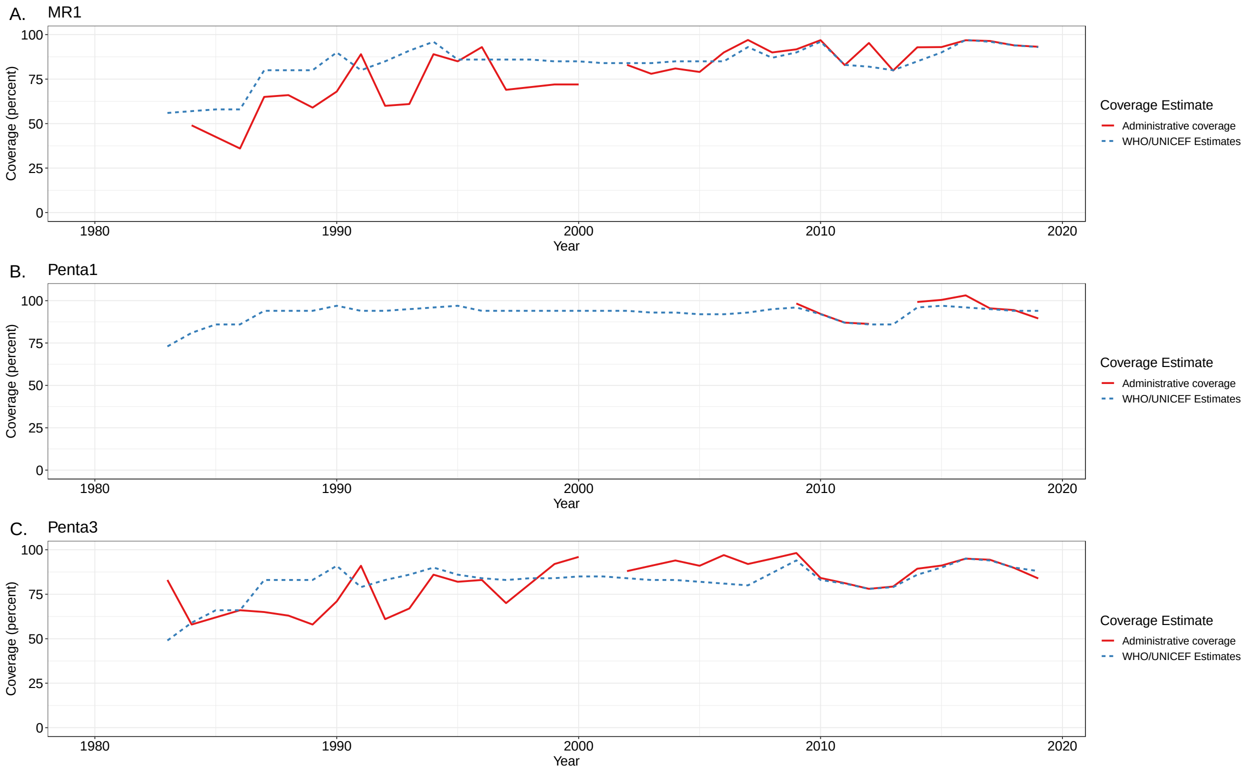

S1 Fig. National level time trends in MR1, Penta1, and Penta3 vaccination coverage in Zambia 1980 – 2019. These data were extracted from the WHO immunization webpage (https://immunizationdata.who.int/).
