# Supplementary material for: An evaluation of the early impact of the COVID-19 pandemic on Zambia’s routine immunization program
Source: PLOS Glob Public Health. 2023 May 2;3(5):e0000554. doi: 10.1371/journal.pgph.0000554 (PMC10153718; doi:10.1371/journal.pgph.0000554)
Supplement: S2 Fig — (PDF) [file pgph.0000554.s005.pdf]

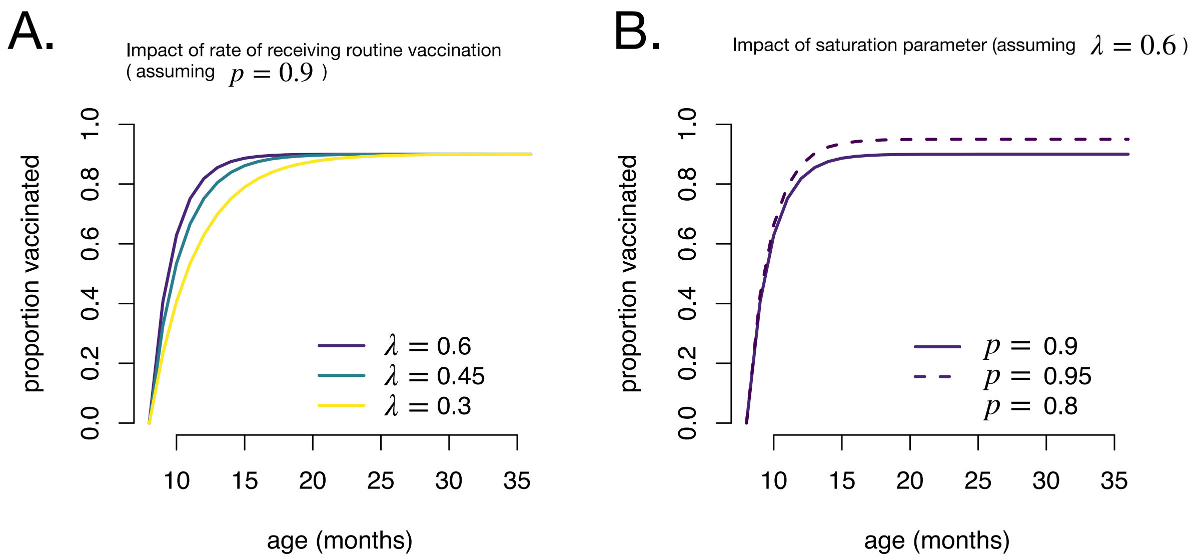

S2 Fig. Impact of estimated parameters λ (**A**) and p (**B**) on the proportion vaccinated over age.
