# Supplementary material for: An evaluation of the early impact of the COVID-19 pandemic on Zambia’s routine immunization program
Source: PLOS Glob Public Health. 2023 May 2;3(5):e0000554. doi: 10.1371/journal.pgph.0000554 (PMC10153718; doi:10.1371/journal.pgph.0000554)
Supplement: S5 Fig — (PDF) [file pgph.0000554.s008.pdf]

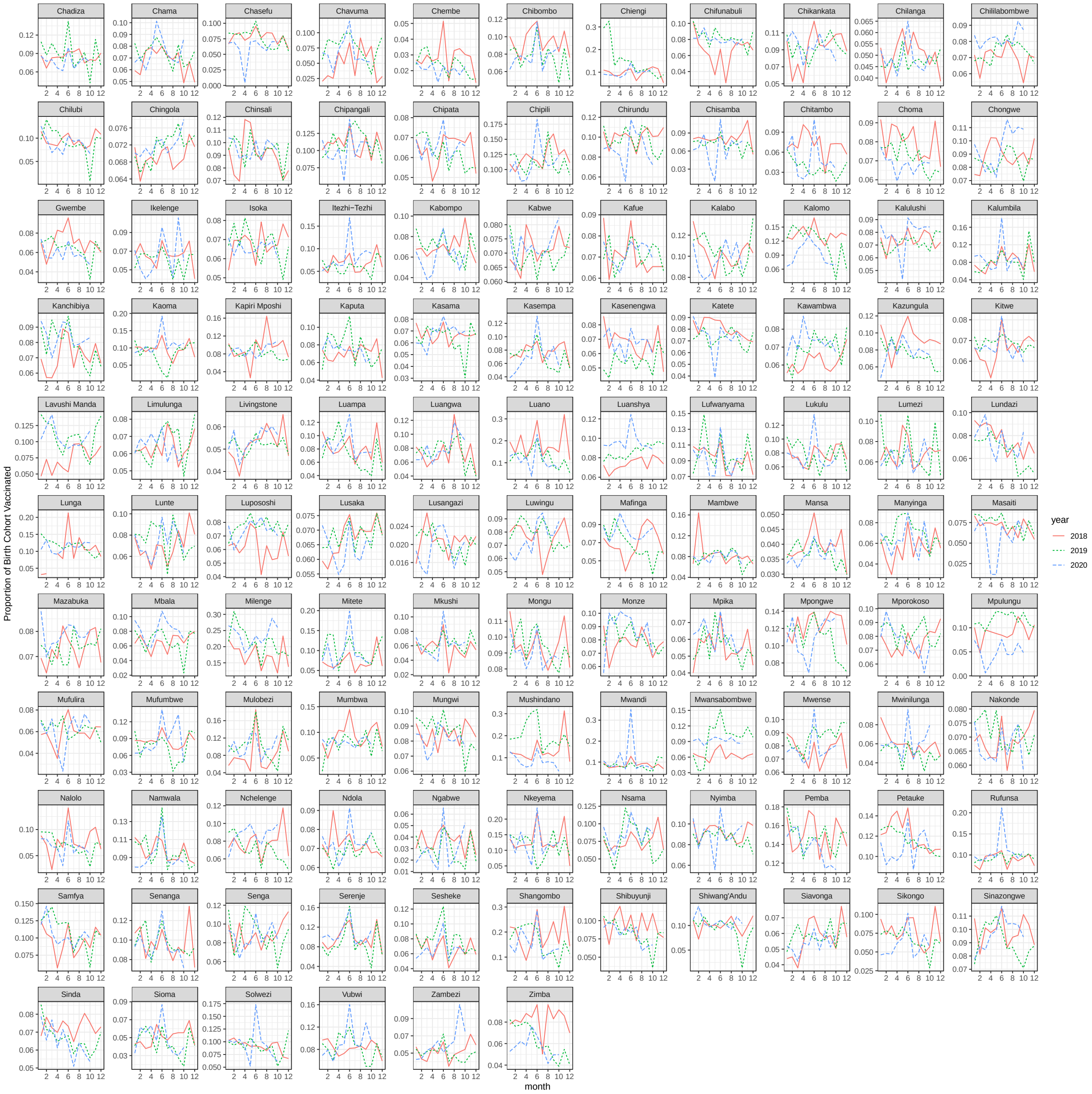

S5 Fig. District-specific administrative Penta3 coverage estimates by month in years 2018, 2019, and 2020. For ease of interpretation we have generated and displayed vaccination coverage estimates rather than the raw number of doses administered. In this plot vaccination coverage was estimated by the reported number of doses administered divided by the size of the birth cohort (i.e., WorldPop population size times the Zambia Central Statistical Office's estimated proportion of the population in the less than one year age group for the respective years). Note the inconsistent patterns across year and months. For many districts (49 of 116) vaccination coverage (i.e., the proportion of a birth cohort to be vaccinated within a year in 2018 & 2019, or within 10 months in 2020) exceeds 100%. We decided these few data points (i.e., two data points for each month and district for pre-pandemic years and one data point for each month and district for pandemic year) were not sufficent for estimating district-specific disruptions. However, we did rely on the variability represented here (i.e., 232 data points for each month for pre-pandemic years, and 116 data points for each month in pandemic year) to estimate national level disruption.
