# Supplementary material for: An evaluation of the early impact of the COVID-19 pandemic on Zambia’s routine immunization program
Source: PLOS Glob Public Health. 2023 May 2;3(5):e0000554. doi: 10.1371/journal.pgph.0000554 (PMC10153718; doi:10.1371/journal.pgph.0000554)
Supplement: S6 Fig — (PDF) [file pgph.0000554.s009.pdf]

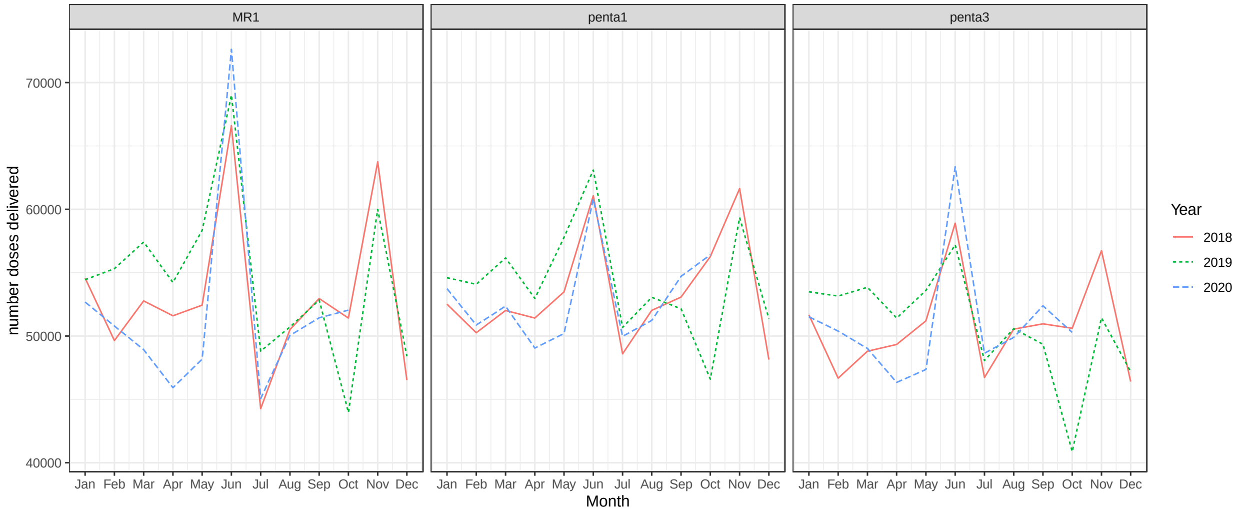

S6 Fig. Raw administrative vaccination data. The number of MR1, Penta1, and Penta3 vaccine doses adminstered from January to December. Each line represents a different year of data. Child health weeks are conducted in June and November of each year, hence the spikes in vaccine doses in these months.
