# Supplementary material for: An evaluation of the early impact of the COVID-19 pandemic on Zambia’s routine immunization program
Source: PLOS Glob Public Health. 2023 May 2;3(5):e0000554. doi: 10.1371/journal.pgph.0000554 (PMC10153718; doi:10.1371/journal.pgph.0000554)
Supplement: S7 Fig — (PDF) [file pgph.0000554.s010.pdf]

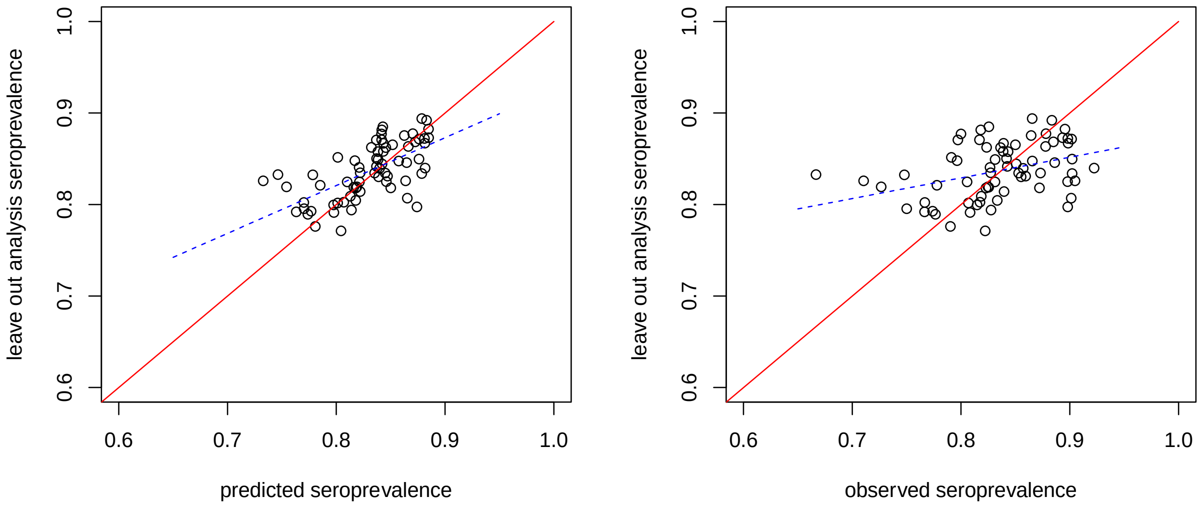

S7 Fig. Results of leave out district analysis. Each point represents a different district. Left figure displays the estimated mean seroprevalence for a predicted districts left out of the analysis by the expected seroprevalence given the district was included in the analysis. Right figure displays the estimated mean seroprevalence for a predicted districts left-one-out of the analysis by the observed seroprevalence for the respective district. Dashed blue line is fit line and red solid line represents perfect agreement.
