# Supplementary material for: An evaluation of the early impact of the COVID-19 pandemic on Zambia’s routine immunization program
Source: PLOS Glob Public Health. 2023 May 2;3(5):e0000554. doi: 10.1371/journal.pgph.0000554 (PMC10153718; doi:10.1371/journal.pgph.0000554)
Supplement: S8 Fig — (PDF) [file pgph.0000554.s011.pdf]

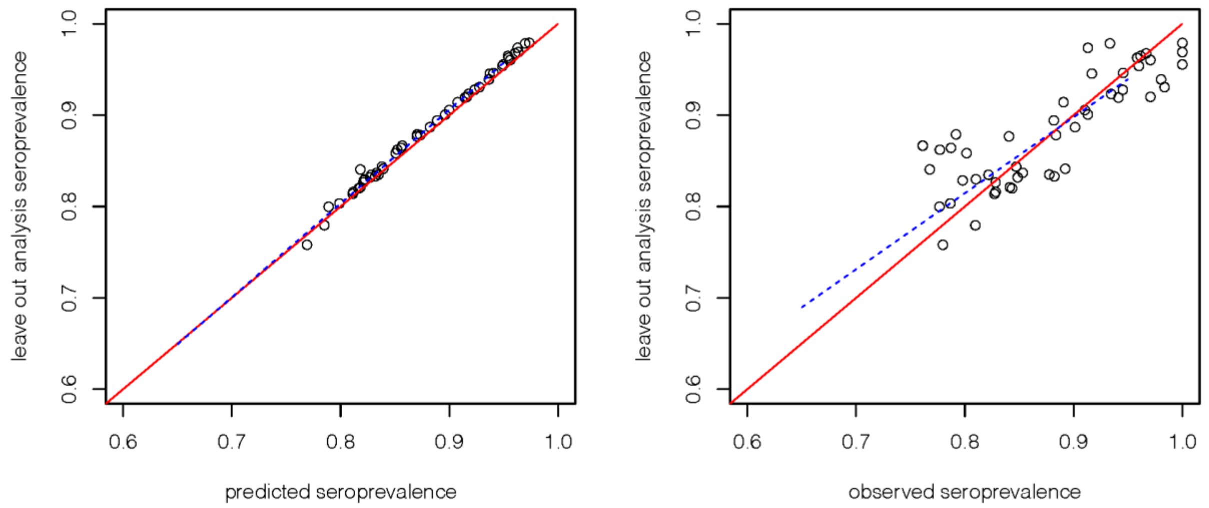

S8 Fig. Results of leave out age analysis. Each point represents a different age in years. Left figure displays the estimated mean seroprevalence for a predicted ages left out of the analysis (y-axis) by the expected seroprevalence given the age was included in the analysis (x-axis). Right figure displays the estimated mean seroprevalence for a predicted ages left-one-out of the analysis (y-axis) by the observed seroprevalence for the respective age (x-axis). Dashed blue line is fit line and red solid line represents perfect agreement.
