# Supplementary material for: An evaluation of the early impact of the COVID-19 pandemic on Zambia’s routine immunization program
Source: PLOS Glob Public Health. 2023 May 2;3(5):e0000554. doi: 10.1371/journal.pgph.0000554 (PMC10153718; doi:10.1371/journal.pgph.0000554)
Supplement: S9 Fig — (PDF) [file pgph.0000554.s012.pdf]

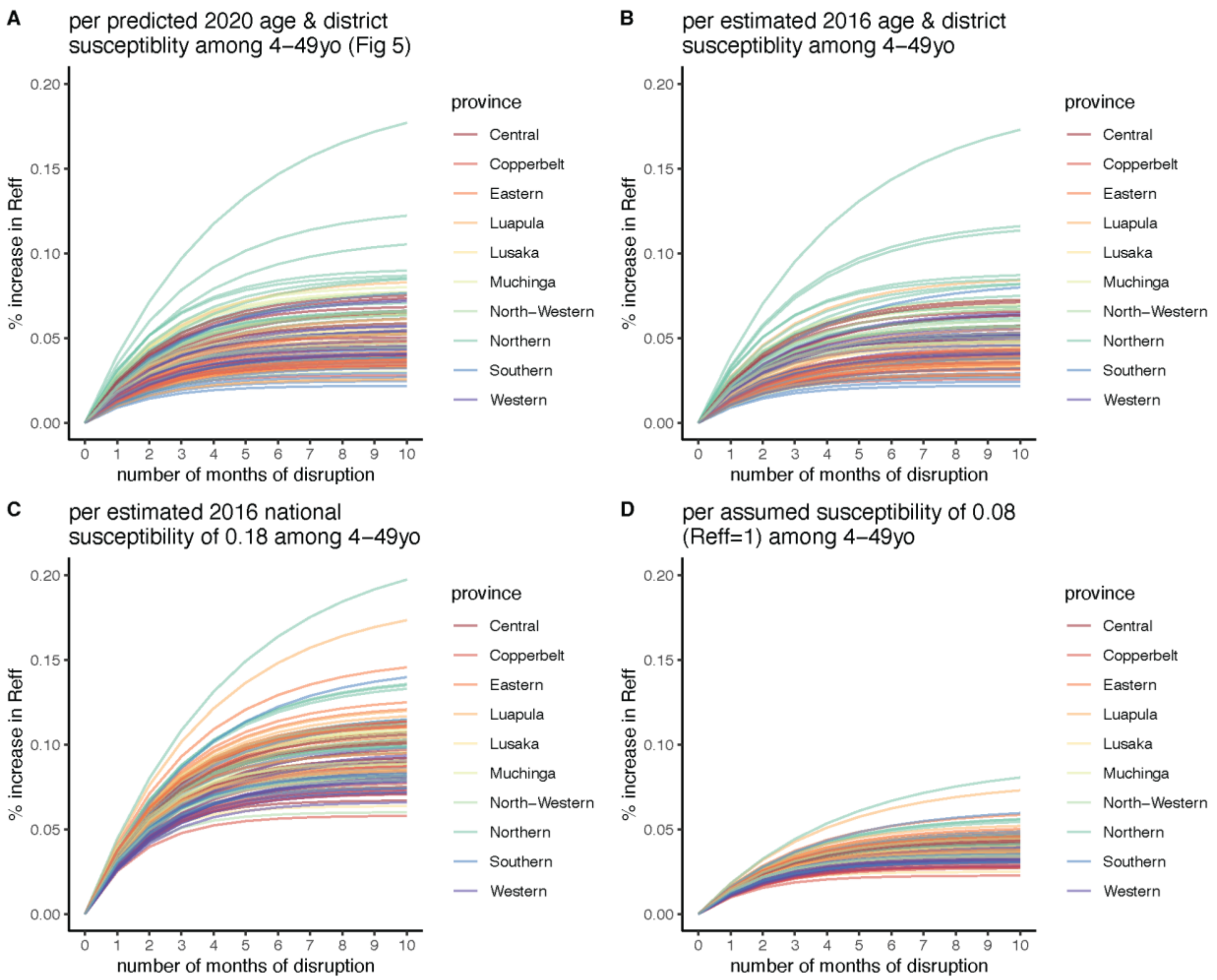

S9 Fig. Sensitivity analysis of percent increase in Reff per month of disruption based on different starting estimates of proportion susceptible across ages 4 to 49 years old. **A)** same as main text Figure 5 is based on predicted 2020 age- and district-susceptibility among 4 to 49 years old. **B)** based on 2016 age- and district-specific susceptibility among 4 to 49 years old. **C)** based on estimated 2016 national susceptibility of 0.18 assumed for all ages 4 to 29 years old. **D)** based on assumed susceptibility of 0.08 (equivalent to Reff=1) assumed for all ages 4 to 49 years old.
