# Supplementary material for: An evaluation of the early impact of the COVID-19 pandemic on Zambia’s routine immunization program
Source: PLOS Glob Public Health. 2023 May 2;3(5):e0000554. doi: 10.1371/journal.pgph.0000554 (PMC10153718; doi:10.1371/journal.pgph.0000554)
Supplement: S11 Fig — (PDF) [file pgph.0000554.s014.pdf]

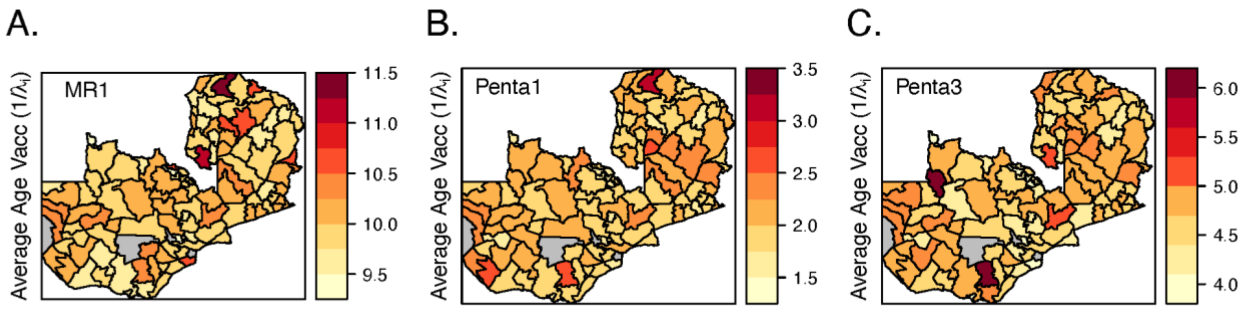

S11 Fig. District-level estimates of the average age of vaccination among those that receive vaccination for MR1 (A), Penta1 (B), and Penta3 (C). Average age is estimated as the inverse of the median rate of receiving routine vaccination. There are no parameter estimates for 4/116 districts colored in grey due to the lack of any DHS sampling clusters in these districts. Shapefile available CC BY 4.0 license via https://data.grid3.org/datasets/GRID3::nsdi-zambia-administrative-boundaries-districts-2022-published-by-grid3/about
