# Supplementary material for: An evaluation of the early impact of the COVID-19 pandemic on Zambia’s routine immunization program
Source: PLOS Glob Public Health. 2023 May 2;3(5):e0000554. doi: 10.1371/journal.pgph.0000554 (PMC10153718; doi:10.1371/journal.pgph.0000554)
Supplement: S14 Fig — (PDF) [file pgph.0000554.s017.pdf]

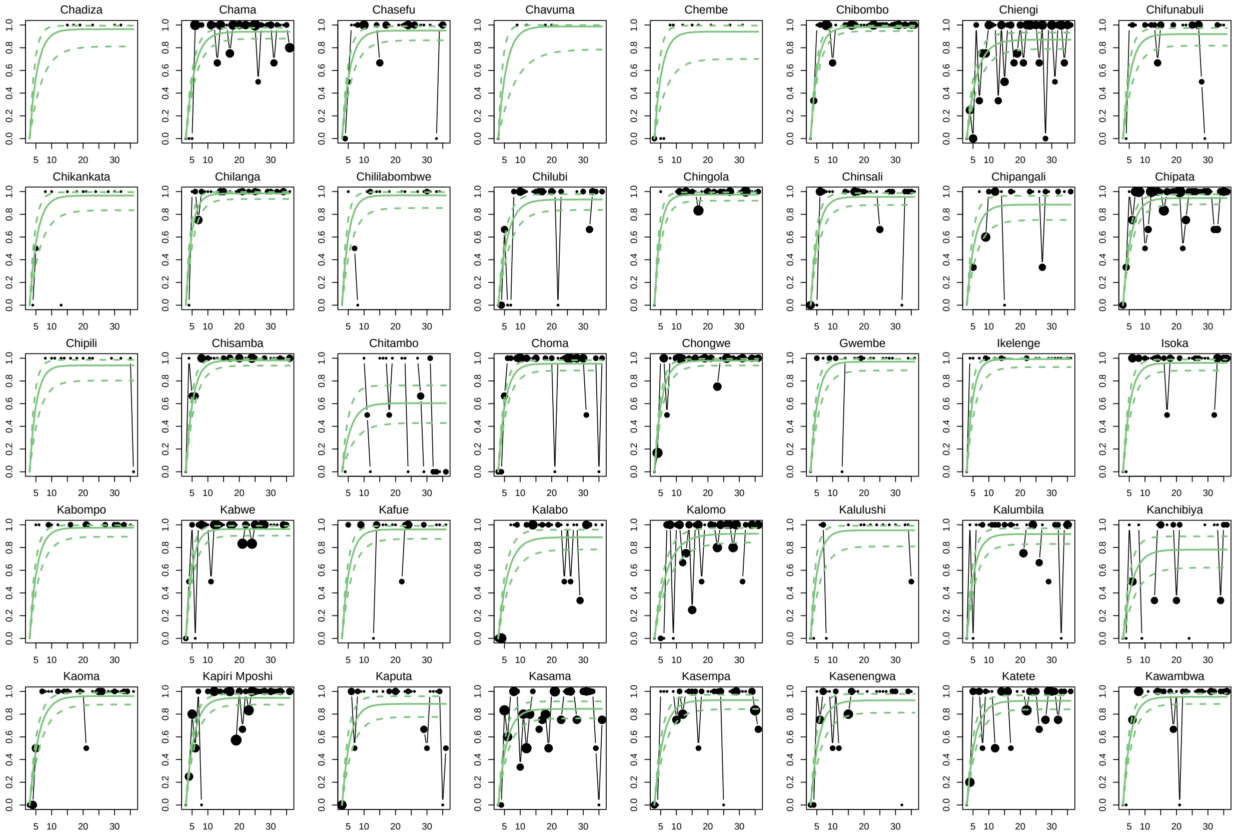
Chadiza
Chama
Chasefu
Chavuma
Chembe
Chibombo
Chiengi
Chifunabuli
Chikankata
Chilanga
Chililabombwe
Chilubi
Chingola
Chinsali
Chipangali
Chipata
Chipili
Chisamba
Chitambo
Choma
Chongwe
Gwembe
Ikelenge
Isoka
Kabompo
Kabwe
Kafue
Kalabo
Kalomo
Kalulushi
Kalumbila
Kanchibiya
Kaoma
Kapiri Mposhi
Kaputa
Kasama
Kasempa
Kasenengwa
Katete
Kawambwa

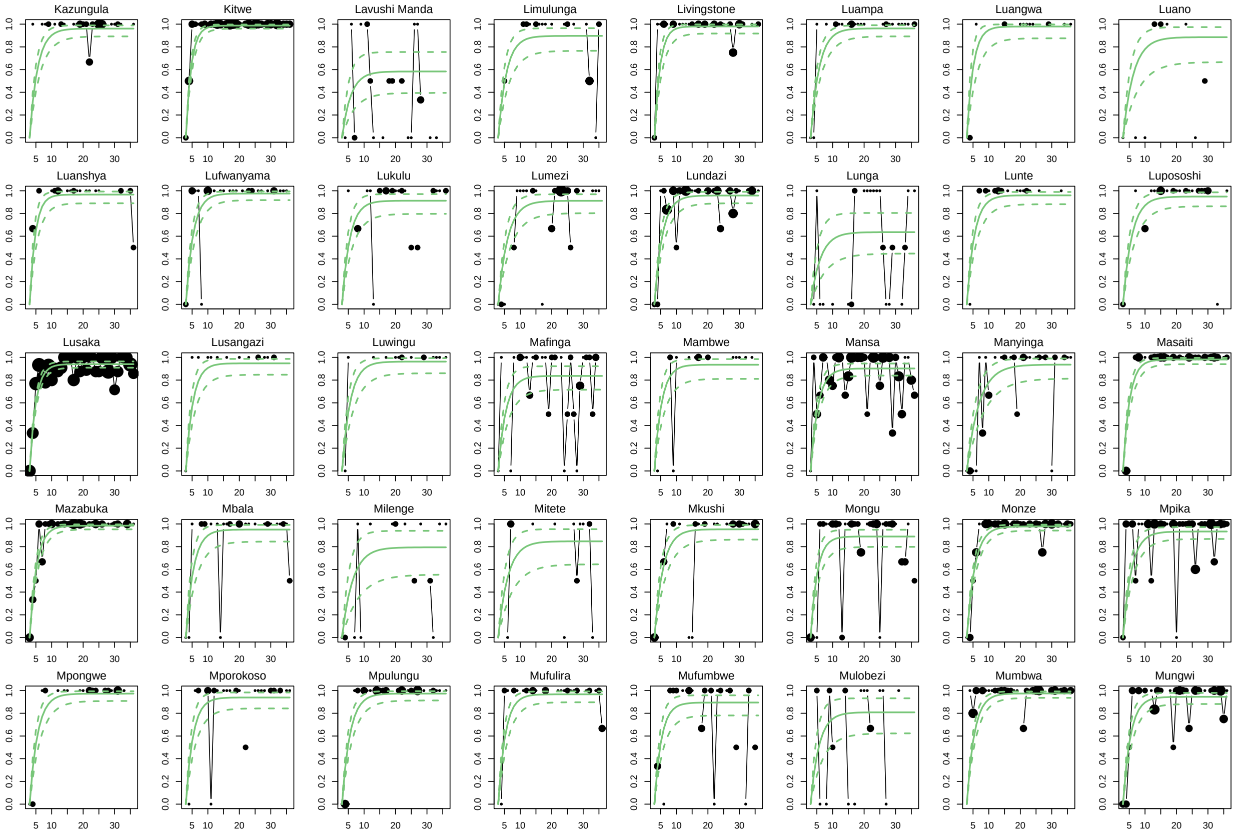
Kazungula
Kitwe
Lavushi Manda
Limulunga
Livingstone
Luampa
Luangwa
Luano
Luanshya
Lufwanyama
Lukulu
Lumezi
Lundazi
Lunga
Lunte
Lupososhi
Lusaka
Lusangazi
Luwingu
Mafinga
Mambwe
Mansa
Manyinga
Masaiti
Mazabuka
Mbala
Milenge
Mitete
Mkushi
Mongu
Monze
Mpika
Mpongwe
Mporokoso
Mpulungu
Mufulira
Mufumbwe
Mulobezi
Mumbwa
Mungwi

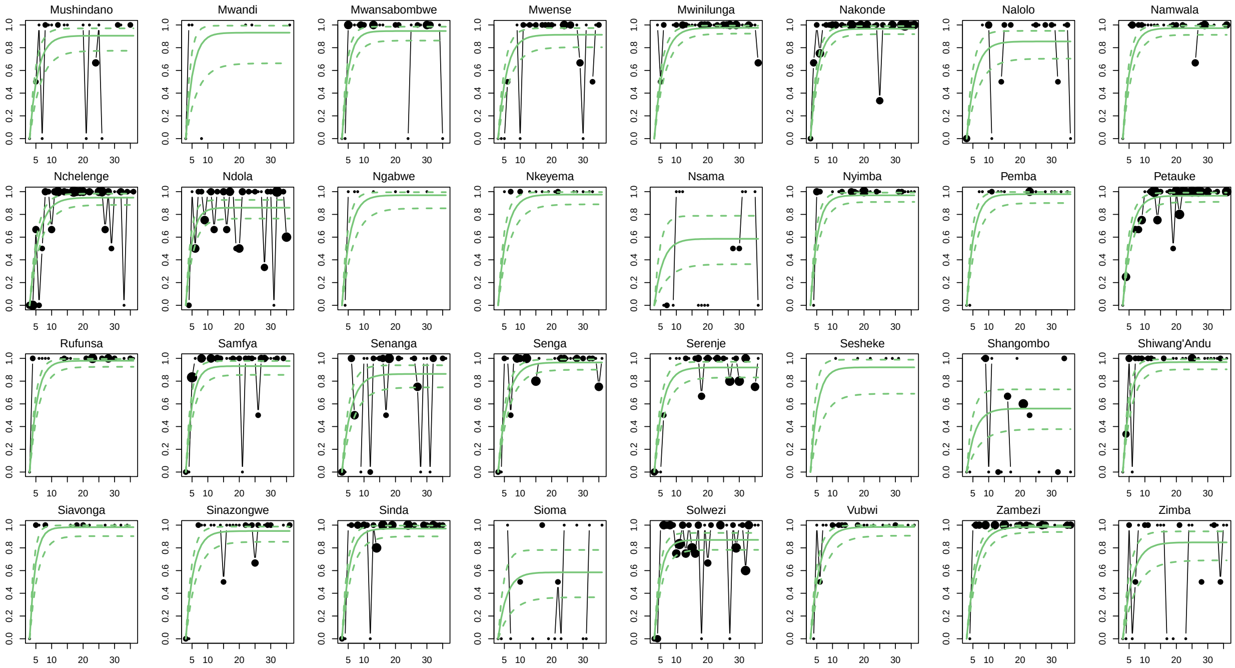

S14 Fig. District-level Penta3 baseline routine proportion vaccinated (y-axis) over age in months (x-axis). Each plot represents one of the 112 modeled districts. The black points represent the data where the size of the point is proportional to the number of observations for each age in months. The solid and dashed lines represent the model fit median and 95% credible intervals.
