# Supplementary material for: An evaluation of the early impact of the COVID-19 pandemic on Zambia’s routine immunization program
Source: PLOS Glob Public Health. 2023 May 2;3(5):e0000554. doi: 10.1371/journal.pgph.0000554 (PMC10153718; doi:10.1371/journal.pgph.0000554)
Supplement: S15 Fig — (PDF) [file pgph.0000554.s018.pdf]

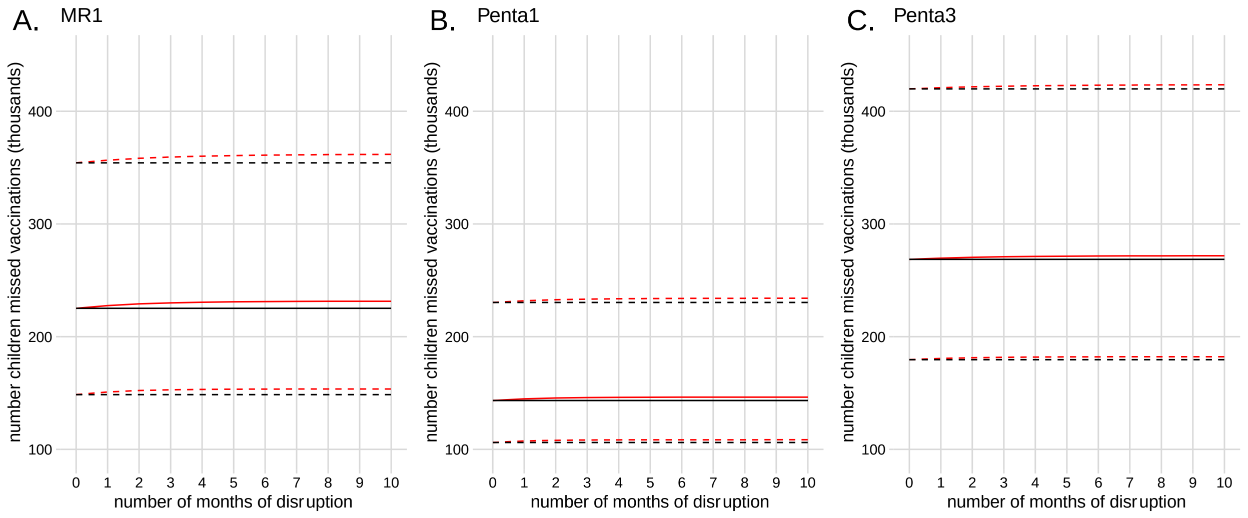

S15 Fig. Estimated number of children missed by vaccination of MR1 (A), Penta1 (B) and Penta3 (C). The solid and dashed black lines represent the median and 95% confidence interval, respectively, of children missed by vaccination in the reference pre-pandemic years 2018/2019. The solid and dashed red lines represent the median and 95% confidence interval, respectively, of children missed by vaccination in the pandemic year 2020 associated with each non-disruption year percentile estimate (median and 2.5% percentile and 97.5% percentile). The estimates associated with the median number of children missed in a reference year are highlighted in Figure 4 of the main text.
