# Supplementary material for: An evaluation of the early impact of the COVID-19 pandemic on Zambia’s routine immunization program
Source: PLOS Glob Public Health. 2023 May 2;3(5):e0000554. doi: 10.1371/journal.pgph.0000554 (PMC10153718; doi:10.1371/journal.pgph.0000554)
Supplement: S10 Fig — (PDF) [file pgph.0000554.s013.pdf]

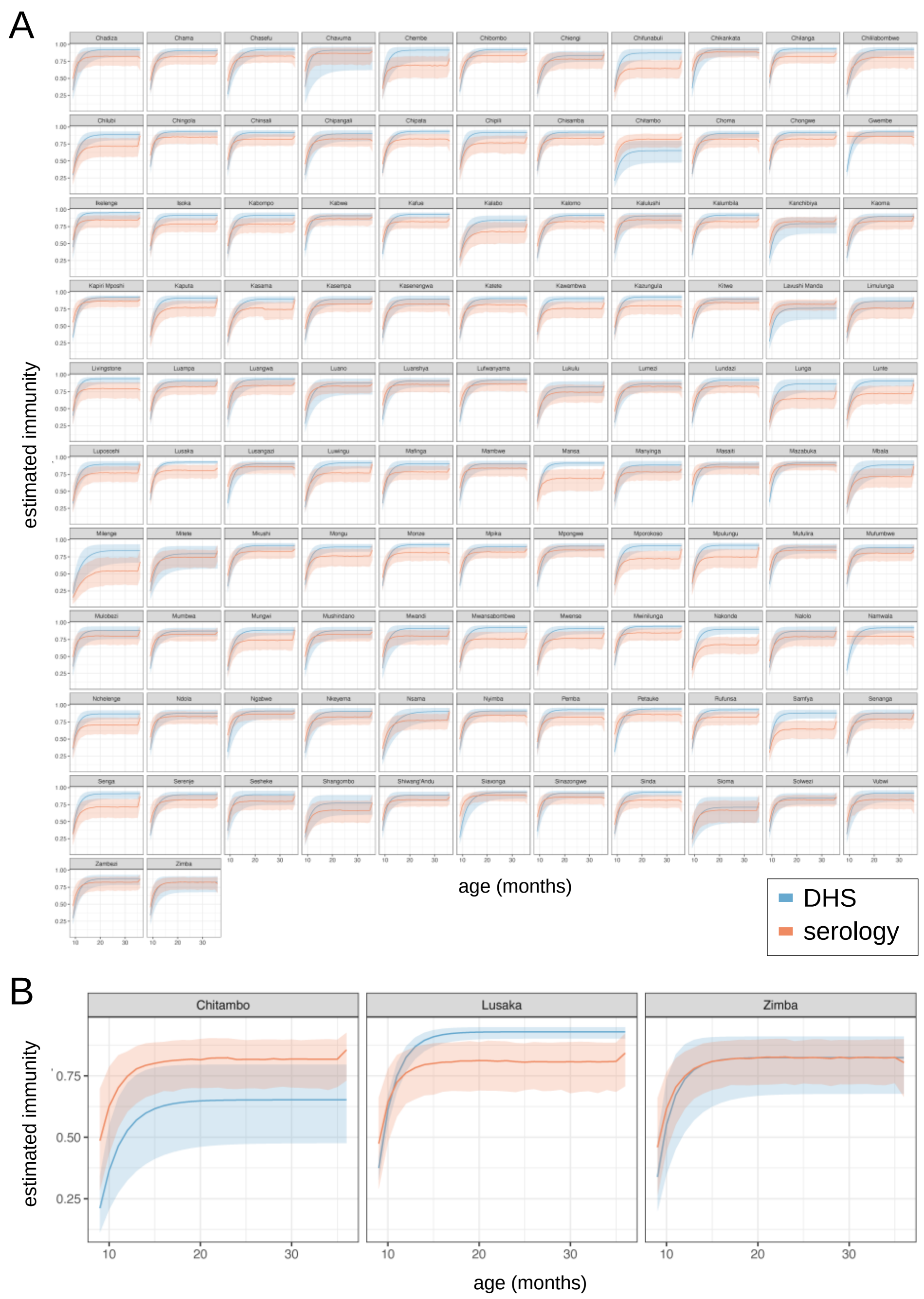

S10 Fig. Comparison of estimated immunity by age (9-36 months) using seroprevalence data and 2018 Zambia DHS data. The described above ‘measles seroprevalence model’ is a hierarchical spatial models fit to individual measles seropositivity in 2016 that was used to predict age and district specific seroprevalence in 2018; the median and 95% credible intervals are displayed in orange line and transparent orange ribbon, respectively. The methods sub-section titled ‘pre-pandemic routine vaccination’ describes a spatial model fit to 2018 DHS vaccination data that was used to estimate age and district specific immunity (taking into account vaccination effectiveness and minimal disruptions); the mean and 95% credible intervals are displayed in blue line and transparent blue ribbon, respectively. **A)** 112 districts, **B)** 3 districts representing serology estimated immunity higher than DHS coverage estimated immunity, (Chitambo), serology estimated immunity lower than DHS coverage estimated immunity (Lusaka), and good agreement (Zimba).
