# Supplementary material for: An evaluation of the early impact of the COVID-19 pandemic on Zambia’s routine immunization program
Source: PLOS Glob Public Health. 2023 May 2;3(5):e0000554. doi: 10.1371/journal.pgph.0000554 (PMC10153718; doi:10.1371/journal.pgph.0000554)
Supplement: Attachment — Submitted filename: ResponseToReviewers.docx [file pgph.0000554.s019.docx]

Reviewer 1:

Thank you for taking the time to review our manuscript. We greatly appreciate your comments and believe they have improved the analysis and readability of the manuscript.

**Major comments**

- Clear time assignments for various statements and reporting. Especially as broader (and certainly country-specific) response to COVID-19 has varied and evolved since March 2020, it is important to be very clear about what time periods (year or month-year) are being discussed and reporting on.
  - Page 2, paragraph 2 (Introduction): The first sentence implies such strong ‘lockdown’ and containment measures are still ongoing worldwide, whereas this really primarily applies in March-April 2020 globally (and then much more country-specific measures were implemented, often in response to more localized conditions)

We revised the sentence by making it past tense and a more general comment of the impact of the pandemic.

- Page 2, paragraph 5 (Methods): it is not completely clear that the disruptions are assessed for each month of July 2020 through January 2021.

Thank you for your comment. We revised the sentence as to the following: “To understand if and how COVID-19 related health care disruptions impacted the risk of vaccine-preventable diseases, we estimated changes in the national routine immunization program during the COVID-19 pandemic year 2020, and its impact on the number of children who missed vaccination (and risk of measles outbreak) at the district level (administrative level 2).” We modified the manuscript by removing the small preliminary analysis of data coverage data November 2020 – March 2021 given reviewers comments and the fact that it was not well motivated.

- Use of 2018 as the baseline for disruptions: while the 2018 year appears beneficial because 2018 estimates from DHS could be derived, it’s not made clear why these data weren’t combined with the 2018 administrative estimates (it appears that the DHS estimates were ultimately used on their own – if this isn’t the case, much clearer descriptions of how each data source was used would be extremely useful) Thank you for your helpful interpretation of our methods section. Given your comments, we substantially revised the methods section to better explain the use of the two different data sets (DHS and administrative data) and why we used each. We also revised figure 1, the methodological road map, to clarify the methods and data. Ultimately, we used DHS data to estimate the rate of vaccination coverage over age by district, aka, the pre-pandemic vaccination rate. We used administrative data to estimate the percent change in the rate of vaccination (using 2018 and now also 2019 admin data vs 2020 admin data). We did not include administrative data estimate our pre-pandemic vaccination coverage rate estimate because these data are considered more unreliable due to incomplete or inaccurate reporting of vaccination, mistake compiling the data across administrative units, and inaccurate population denominator estimates. Survey data, while it still has it challenges, are generally of higher quality and in the least have a better grasp of the denominator (i.e., number of children surveyed). Therefore, we only used administrative data to evaluate time trends or the change in vaccination based on the assumption that biases in these data are consistent across time.

Furthermore, potentially even more importantly, it was never discussed why 2019 was completely omitted from baseline estimation approach. Presumably whatever potential biases between administrative data and survey data could be calculated based on the shared year of data availability (2018) and bias adjustments could then be applied to 2019 administrative data. In addition, it’s possible that 2018 involved district-time deviations (e.g., stockouts, heightened vaccination administration in relation to a campaign or boost of funding) from ‘a true baseline’ that would then affect the relative disruption estimated for 2020. Including an additional year for establishing an ‘average’ baseline would help identify those potential issues. At minimum, not talking more about the implications of omitting 2019, which provides a closer temporal baseline to 2020 disruptions, and why authors didn’t consider using both 2018 and 2019 as baseline indicators seems like a pretty substantial omission. Thank you for your suggestion. We have incorporated the 2019 administrative estimates to the analysis. Specifically, we now use the 2018 and 2019 administrative vaccination data as “baseline” compared to 2020 administrative vaccination data to estimate a percent change in the rate of vaccination as of 2020.

- Inclusion of the 2020-2021 disruption analysis in the Methods section: this is added in on page 7, paragraph 2, but involves what could be an additional dataset and estimation approach (e.g., it doesn’t seem that 2018 is used as the baseline?). This secondary (?) analysis needs to be clearly explained in the Methods and then linked to the Results. Thank you for your comment. We added this preliminary analysis in last minute because our Zambian collaborators were interested. For the sake of the paper, it was not well motivated, our apologies. We have removed this from the analysis.
  - The minimal or no disruptions in routine vaccination services takeaway seems to be contradictory to the MCV1 results (a mean reduction of 3.8% [95% CI 2.1 to 5.3%] for MCV1 does not appear to be minimal or a non-disruption).
- Subnational analysis based on methodological framework presented in this study. The paper emphasizes the use and application of its analytical approach to understanding subnational pandemic-related disruptions; however, national mean percent reductions in routine disruptions were ultimately applied to district-level baseline estimates to produce estimates of district-level disruptions. This is a good point, and we greatly appreciate you highlighting this issue. We have substantially revised the language of the paper to reflect the paper’s contribution more accurately, specifically assessing the impact of national disruptions on the number of missed children at the subnational level. In application, subnational disruptions likely varied from this national mean, and with the data presented, it seemed as if at least some kind of subnational disruption ratio would be calculatable (or at least provincial [admin1] estimates if district-level [admin2] was not possible). The authors should either provide more caveats around how this methodological framework actually can capture subnational disruptions (and recovery), present sensitivity analyses about why they ultimately didn’t use subnational disruption ratios and why it was better to assume a national percent reduction, or both. We have added three new supplementary figures (S2-S4) displaying the district-specific administrative coverage data. In the caption of each supplemental figure, we discuss some of the data quality issues, sample size restraints, and reason we decided to estimate national level disruptions.
  - Page 7, Discussion, paragraph 1: “We performed a detailed analysis of the impact of the COVID-19 pandemic on the routine immunization program in Zambia across time (by month) and space (at administrative level 2). The methodological framework developed in this analysis can be used to evaluate COVID-19 pandemic disruptions in other countries or for other vaccines at the subnational level.” *This framing makes it sound like all components of this analysis was, including estimation of disruptions, occurred at the subnational level rather than assuming that national-level disruption rate was what occurred at the district-level.* This sentence has been revised to say the following, “We performed a detailed analysis to estimate changes in the national routine immunization program in Zambia during the pandemic year 2020, and its impact on the number of missed children (and risk of measles outbreak) at the district level (administrative level 2). The methodological framework developed for this analysis can be used to estimate COVID-19 pandemic disruptions and its impact at the subnational scale in other countries or for other vaccines.”
  - Page 8, Discussion, paragraph 2: “The result is that potential heterogeneities in disruptions to routine services over space were not captured.” This is a really important limitation that affects how the ways this methodology and analytic approach should be framed – the study’s approach enables the use of subnational data to quantify national level estimates of disruption, but it has yet to be used to quantify how this truly happens over space. To continue emphasizing the use of this approach, as is it currently stands, as an analysis of subnational disruptions could risk being very misleading.
    - “Importantly, this analysis suggests that variation in the number of unvaccinated children in a non-disruption year was much larger than the variation in unvaccinated children during the COVID-19 pandemic disruptions in 2020. This finding further highlights the minimal impact of the COVID-19 pandemic on routine immunization services in Zambia.” <- this kind of conclusion doesn’t seem supportable by this analysis – especially when a national disruption rate was applied to district level baseline rates. Without being able to understand subnational disruption rates, it doesn’t seem possible to appropriately say that it varied less – and thus there was minimal disruptions – during 2020 (especially compared with the huge limitation discussed 2 sentences prior). We have revised the text. It now reads, “administrative vaccination data was not sufficient to estimate sub-national disruptions to routine vaccinations, so we estimated a national rate of reduction with uncertainty based on district level data. Variation in the number of unvaccinated children across districts was driven by district-specific size of birth cohorts and pre-pandemic district-specific vaccination coverage and did not include the heterogeneities in disruptions to routine services over space. As a result, the variation in the number of unvaccinated children is likely smaller than truly exists and should be interpreted through this lens.”
- Framing around results as having little or minimal impact on RI services. Given data limitations and the observational nature of this analysis, it is strongly recommended to temper statements around the COVID-19 pandemic having little or minimal impact on routine immunization services and childhood vaccination in Zambia. Some of these findings may be artefacts of using the 2018 DHS as the baseline data source (versus 2019 or another year), as well as model assumptions (e.g., national mean disruption applied to district-specific baselines). This means using less strong and conclusive language, which shouldn’t fundamentally change the main takeaways – instead, it more accurately represents what the analysis can support. Examples:
  - Page 1, Conclusions (Abstract):
    - Current: “The pandemic only minimally increased the number of children missed by measles-rubella and pentavalent vaccines in 2020.”
    - Recommended: “This study estimated minimal increases in the number of children missed by vaccination services in Zambia during 2020.”

Thank you for your suggested revised text. We have adopted these changes into the paper.

- Page 8, Discussion, paragraph 3:
  - Current: “Despite documented impact of the pandemic on vaccination programs in many countries (2), we found minimal disruptions to the ability of the routine immunization program in Zambia to deliver MR1, Penta1, and Penta3 vaccines between January 2020 and March 2021 compared to pre-pandemic levels.”
  - Recommended: “Compared to 2018, this study estimated minimal pandemic-related disruptions to childhood vaccination in Zambia in 2020 and early 2021.”

Thank you for your suggested revised text. We have adopted these changes into the paper.

**Minor comments**

***Abstract***

- Page 1, Methods:
  - What does “N=5,670” refer to here? The number of children estimated for 2020, or the number of total data points for between DHS and administrative data? It refers to DHS sample size. We have removed the reference to the sample size in the abstract given the confusion and word count limitations.
  - Name sources of the routine vaccination data (DHIS2?) and serological data used for the measles-specific analysis. The administrative data was obtained from our EPI program collaborators who also contributed as co-authors on the paper. We added detail about the serosurvey but feel that administrative vaccination data is a sufficient explanation for the abstract.

***Introduction***

- Page 2, paragraph 1: cite an amount of confirmed cases as of a given date, instead of “hundreds of millions” revised
- Page 2, paragraph 1: additional factors contributing to potential disruptions included reduced capacity/service offerings in general, restrictions on movement under stay-at-home orders and like COVID-19 mitigation measures. Consider revising to parental/caregiver reluctance versus solely maternal reluctance. Additional citations to consider to support these statements include:
  - UNICEF, May 2020: <https://www.unicef.org/press-releases/least-80-million-children-under-one-risk-diseases-such-diphtheria-measles-and-polio>
  - Gavi situation reports: <https://www.gavi.org/news-resources/document-library/covid-19-situation-reports>
  - Dixit et al 2021: <https://gh.bmj.com/content/6/7/e005031>

Thank you for your suggested additions. We have revised the text to include these factors and added the supporting references.

- Page 2, paragraph 2: had 68 countries fully suspended vaccination services, or had some kind of kind of reduced service provision? Further, 90 mass campaigns canceled or postponed for MR alone or all previously planned mass campaigns? We revised the sentence to read “According to the World Health Organization (WHO) the suspension or disruption of vaccination services in over 68 countries during the early stages of the pandemic put over 80 million infants younger than one year of age at risk of measles (6).” We additionally added some additional context to this paragraph of the introduction to better reflect our knowledge today. Specifically, “While countries are working hard to maintain optimal vaccination program performance, we have started to see the result of some of these disruptions. For example, in 2022 after years of having eliminated wild poliovirus, Malawi and Mozambique reported an outbreak of wild poliovirus and wild poliovirus cases have popped up in the United Kingdom and the United States of America (9). Additionally, the World Health Organization has warned of “perfect storm” condition for measles outbreaks given measles high transmissibility, pandemic-related disruptions, and displaced populations due to conflicts or crises (11).”
- Page 2, paragraph 3: “difference to the pandemic” – consider revising to “pandemic-related disruptions” Thank you for your suggestion. One of the other reviewer’s comments was a revision of the language to be clear this is not a causal analysis. You may notice as a result, some of the language has been changed, including this sentence.

***Methods***

- Page 2, paragraph 4: considering using this citation around why DTP3/penta3 is used as an indicator, and revise to immunization program performance (rather than healthcare):
  - <https://academic.oup.com/jid/article/224/Supplement_4/S452/6378083>

Great, thank you. Citation and text revised as suggested.

- Page 3, paragraphs 2 and 4: confirm consistency in districts at this time (n=115 versus 116).

Prior to our submission in March 2022, the Central Statistical Office in Zambia had only made available a shapefile with 115 districts. As of November 2022, GRID3 published a shapefile with all 116 districts. We have updated all the analyses to include all 116 districts (although there are still four missing from the DHS estimates), and revised text as appropriate.

- Page 3, paragraph 3: consider adding “in 2020” for the magnitude of disruption to ensure that readers understand the time frame in which you’re quantifying the disruptions

“in 2020” was added

- Page 4, paragraph 2: how uncertainty was incorporated is fairly confusing (lower bound: subtracting the 2.5^th^ quantile of the 2018 month disruptions from 97.5^th^ quantile from 2020 month estimates; upper bound: subtracting 97.5^th^ quantile from 2018 month estimates and 2.5^th^ of the 2020 estimates). Is there a reason why a more formal simulation process (e.g., bootstrapping) wasn’t used to then estimate the uncertainty for the mean value of interest (national-level mean)? We revised our estimate of uncertainty bounds using the posterior distributions of the rate of vaccination for each time period (pre-pandemic 2018-2019 and pandemic 2020) and each vaccine (MR1, Penta, and Penta3).
- Page 4, paragraph 4: what 10 months comprised the national mean? We revised the text to specify January 2020 – October 2020.

***Results***

- In general: since there are so many different analyses occurring in this paper, the results reporting could be substantially strengthened by clearly organizing the results section by analytic section. Otherwise, it gets very confusing with the different time points under analysis.

Thank you for your suggestion, we have added subheadingsxs to the results that are consistent with the methods subheadings.

- In general: please be consistent in reporting significant digits. On page 6, paragraph 3, specific digits for hundreds, tens, and ones were reported whereas rounded values (e.g., 23,000 children, about 320 children) were reported in paragraph 4 of the same page. Thank you for your suggestion, we have revised all numbers to report to the second decimal place.
- Page 5, paragraph 5: confirm that the range for MR1, Penta1, and Penta3 is the full range (min-max) or IQR. Yes, it is the full range (min and max).
- Page 6, paragraph 1: “Districts with a lower rate of vaccination for MR1 did not necessarily have a lower rate of vaccination for Penta1 and Penta3” – is this for the baseline year? Or another time period? This is based on pre-pandemic DHS 2018 data. With the new subheadings, this should now be clear.
- Page 6, paragraph 2: “an increase in the proportion of the annual birth cohort in the month of June because of Zambia’s Child Health Week” – which year? Or all years? And relative to which month – May or the start of the year? And what about the effects of the December Child Health Week (i.e., usually child health weeks happen twice a year in Zambia)? Increase in June comparing the pre-pandemic years (2018/2019) to the disruption year (2020). We revised the text to specify the comparison, it now reads “For all three vaccines, we found an increase in the proportion of the annual birth cohort vaccinated in the month of June (2018/2019 compared to 2020) because of Zambia’s Child Health Week (Fig. 3).” The second child health week in 2020 occurred in November, in tandem with the MR campaign. Our analysis stopped as of October 2020.
- Page 7, paragraph 1: is the ‘increase’ in the measles R(e) actually statistically significant (or more importantly, scientific meaningful)?

We did not perform a statistical test on the change in R effective, given its calculation did not include variation and was based on the mean estimated age-specific susceptibly and the age contact matrix. We added text to the discussion regarding the epidemiologic significance.

- Figure 4: Please round the numbers and put on a gradient rather than deciles (?); this should also have grayed out districts (n=4 or 5? By 2020, I believe there are were 116 districts in Zambia) for which estimates were not generated.

We revised the figure legend to include a color gradient. Thank you for your comment, it improved the readability of the figure.

**Discussion**

- In general: authors should consider restructuring the Discussion section to follow a more typical format: summary paragraph, 1-3 paragraphs on interpreting the results and relating to the current literature, 1 paragraph on limitations (which currently follow the summary paragraph?), and a conclusion paragraph. It would substantially strengthen the takeaways and key considerations for future analyses. We completed revamped the discussion section to follow a more typical format.
- In general: it might be worth mentioning how the study’s estimates compare with another paper’s findings (which is cited). For instance, Causey et al 2021 estimated a 0.2% disruption attributable to the COVID-19 pandemic for DTP3 and 0.5% for MCV1 for 2020: <https://ars.els-cdn.com/content/image/1-s2.0-S0140673621013374-mmc1.pdf> Thank you for highlighting these estimates by Causey et al, we have included a reference to them and found our estimates were well aligned.
- Page 8, paragraph 4: has there been additional documentation in Zambia, or nearby countries, about the potential positive and negative effects around pandemic-related disruptions? For instance, polio occurring in Malawi and Mozambique in 2022 after years of having eliminated wild polio transmission highlights what could emerging after the more acute effects of pandemic-related ‘shutdowns’ and disruptions in 2020. <https://www.voanews.com/a/who-concerned-over-polio-outbreak-in-southeastern-africa/6579203.html> Thank you for pointing us to this article, we have incorporated it into the discussion section regarding the potential post-analysis continued impact of the pandemic.
- Page 9, paragraph 3: are the there examples or citations that can be included for a time-specific seroprevalence model? Not that I am aware of, at least in the way I described it. In the revamping on the discussion section, we decided to remove this sentence given it is not backed up by published literature.
- Page 9: please include a more formal conclusions paragraph, summarizing the study’s main takeaways and implications. The current last paragraph is better suited for the interpretation and contextualization sections of the Discussion. Thank you for your comment, we have added a more formal conclusions paragraph.

Reviewer #2:

Major comments

Regarding “As it is difficult to establish a causal link between the pandemic and vaccination programs, here we compared pre-pandemic and pandemic vaccination program performance and attributed the difference to the pandemic” and “The disruption rate per month was estimated as the percent reduction between the 2018 and 2020 in monthly estimates”:

Were immunization rates already increasing or decreasing prior to the pandemic? The authors can use data from previous DHS surveys to assess this

- If stable before the pandemic, that’s ideal and validates an assumption of this study

 - If already decreasing before the pandemic, need to argue for why observed decreases are the effect of the pandemic and not due to exogenous causes.

- If already increasing before the pandemic, an observation of no change between 2018 and 2020 would be a potentially important finding, indicating that the pandemic potentially inhibited further increases. In this case, I’d recommend a difference-in-differences analysis to quantify the effect size.

Generally needs better discussion of potential time-varying confounders.

Thank you for your comment. We added an additional supplemental figure (S1-S3) showing the time trend of MR1, Penta1, and Penta3 coverage. We find that MR1 and Penta1 generally stabilized prior to the pandemic, while Penta3 was slightly declined in 2018 and 2019. We also added text to the discussion section addressing the potential for time-varying confounders. Lastly, we revised the language throughout the manuscript to make sure not to use causal language.

  --

The authors assessed change over time using the number of doses administered in 2018 and 2020, respectively. By using this metric as a proxy for immunization rates, it is implicitly assumed that the population denominator is constant over time.

This seems unlikely to be true given Zambia’s generally large increase in population in recent years, partially driven by improvements in child survival which would increase the size of the child population. If the child population is indeed increasing and not captured in this analysis, this would lead to upward bias in change in immunization rates over time

- Do WorldPop data (“the size of the population in the district i estimated by aggregating WorldPop population estimates in 10x10 km grid cells over district”) have enough time granularity to capture this effect?

- If not, I encourage the authors to discuss this potential bias in the Discussion section or attempt to adjust for it quantitatively

We allowed the denominators to change over time as well using WorldPop annual population estimates. Thank you for your question as we revised the text in the methods to be clearer.

  --

Minor comments

Regarding “the lower range of the disruption rate per month was estimated as the percent difference between the 2.5th quantile of the 2018 month estimates and the 97.5th quantile of the 2020 estimates”: This seems backwards to me; 2.5th quantile in 2018 should be compared to 2.5th quantile in 2020. Or if my comment is incorrect, I would like to see a better explanation for the method of quantifying uncertainty.

We revised the uncertainty estimates making use of the posterior distributions of our estimated rate parameter. The methods section now reads, “We estimated the disruption per month as the percent reduction in 2020 vaccination rate (compared to 2018/2019 vaccination rate) based on 2,000 draws of the 2020 posterior distribution of $\gamma_{t}$ and 2,000 draws of the 2018/2019 posterior distribution of $\gamma_{t}$. We calculated the mean and 95% uncertainty intervals of the precent reduction each month *t*. Given monthly variation, we then calculated the mean across 10 months (January 2020 – October 2020) to generate a national mean and 95% uncertainty interval of the percent reduction in routine rate for each vaccination dose, and assumed it was constant into the future”

--

Regarding “We found minor disruptions to routine administration of measles-rubella and pentavalent vaccines in 2020. This was in part due to Zambia’s Child Health Week held in June of 2020 which helped to reach children missed during the first six months of the year”: What exactly was due to the Child Health Week (CHW)? This reads as “disruptions” were due to CHW, but I think the authors mean the minor-ness of the disruption was due to CHW. Please re-word.

You are correct. Our apologies for the typo, we revised the text.

--

  The analysis assumes that the sampled populations in the pre- and post-pandemic periods are not systematically different. The authors should argue for why they think this assumption holds in spite of many societal-level factors changing in an unprecedented way during the study period.

We have added a discussion about time-varying covariates.

  --

  The section assessing measles risk is a valuable addition to the study and appears well-conducted.

Thank you.

  --

Regarding "However, it is difficult to compare the number of children who received vaccination in November of 2020 to November of 2018 because the target population is inconsistent between the two years (2018 9-23 months, 2020 9-59 months); therefore, the month of November was excluded from the disruptions estimates between November 2020 and March 2021 for MR1." - Discarding data is a drastic step and I do not fully understand why it was necessary. 9-59 months is a superset of 9-23 months, so the two periods seem compatible me. Please elaborate. - Please provide a sensitivity analysis showing the results with and without the discarded November data.

Thank you for your comment. We added this preliminary sub-analysis in last minute because our Zambian collaborators were interested. For the sake of the paper, it was not well motivated, our apologies. We have removed this sub-analysis from the paper.
